# Statin-induced microRNAome alterations modulating inflammation pathways of peripheral blood mononuclear cells in patients with hypercholesterolemia

**DOI:** 10.1042/BSR20201885

**Published:** 2020-09-21

**Authors:** Hung-Ju Lin, Sung-Liang Yu, Ta-Chen Su, Hsiu-Ching Hsu, Ming-Fong Chen, Yuan-Teh Lee, Kuo-Liong Chien, Tzu-Pin Lu

**Affiliations:** 1 Department of Internal Medicine, National Taiwan University Hospital and College of Medicine, Taipei, Taiwan; 2 Department of Clinical Laboratory Sciences and Medical Biotechnology, College of Medicine, National Taiwan University, Taipei, Taiwan; 3 Centers of Genomic and Precision Medicine, National Taiwan University, Taipei, Taiwan; 4 Departments of Internal Medicine and Environmental and Occupational Medicine, National Taiwan University, Taipei, Taiwan; 5 Institute of Occupational Medicine and Industrial Hygiene, National Taiwan University College of Public Health, Taipei, Taiwan; 6 Cardiovascular Research Laboratory, Cardiovascular Center, Clinical Outcome Research and Training Center, Big Data Center, China Medical University Hospital, China Medical University, Taichung, Taiwan; 7 Institute of Epidemiology and Preventive Medicine, Department of Public Health, College of Public Health, National Taiwan University, Taipei

**Keywords:** hypercholesterolemia, inflammation, microRNA, peripheral blood mononuclear cells, statins

## Abstract

Statins inhibit cholesterol biogenesis and modulate atheroma inflammation to reduce cardiovascular risks. Promoted by immune and non-immune cells, serum C-reactive protein (CRP) might be a biomarker suboptimal to assess inflammation status. Although it has been reported that statins modulated inflammation via microRNAs (miRNAs), evidence remains lacking on comprehensive profiling of statin-induced miRNAome alterations in immune cells. We recruited 19 hypercholesterolemic patients receiving 2 mg/day pitavastatin and 15 ones receiving 10 mg/day atorvastatin treatment for 12 weeks, and performed microarray-based profiling of 1733 human mature miRNAs in peripheral blood mononuclear cells (PBMCs) before and after statin treatment. Differentially expressed miRNAs were determined if their fold changes were >1.50 or <0.67, after validated using quantitative polymerase chain reaction (qPCR). The miRSystem and miTALOS platforms were utilized for pathway analysis. Of the 34 patients aged 63.7 ± 6.2 years, 27 were male and 19 were with coronary artery disease. We discovered that statins induced differential expressions of miR-483-5p, miR-4667-5p, miR-1244, and miR-3609, with qPCR-validated fold changes of 1.74 (95% confidence interval, 1.33–2.15), 1.61 (1.25–1.98), 1.61 (1.01–2.21), and 1.68 (1.19–2.17), respectively. The fold changes of the four miRNAs were not correlated with changes of low-density-lipoprotein cholesterol or CRP, after sex, age, and statin type were adjusted. We also revealed that RhoA and transforming growth factor-β signaling pathways might be regulated by the four miRNAs. Given our findings, miRNAs might be involved in statin-induced inflammation modulation in PBMCs, providing likelihood to assess and reduce inflammation in patients with atherosclerotic cardiovascular diseases.

## Introduction

Atherosclerosis is an arterial inflammatory process of atheroma plaque formation initiated by the erroneous accumulation of apolipoprotein B-containing cholesterol, specifically low-density lipoprotein cholesterol (LDL-C), in subendothelial space of arteries [1]. Atherosclerotic cardiovascular disease (ASCVD) is the leading cause of death worldwide, resulting in nearly 12-million deaths from ischemic heart disease, ischemic stroke, and peripheral arterial disease worldwide [2]. Hypercholesterolemia is one of well-known cardiovascular risk factors, and the risks for coronary heart disease would be raised by 26% and 23% per 1 mmol/l cholesterol increase, in men and women, respectively [3]. Known as an efficacious treatment for hypercholesterolemia, statins lower the risk for major vascular events by 23% per 1 mmol/l LDL-C reduction [4].

Statins have been suggested to exert anti-inflammatory effects on immune cells [5]. The immune cells involved in inflammation progression and resolution of atheroma plaques are mainly from peripheral blood mononuclear cells (PBMCs), including monocytes, lymphocytes, and dendritic cells [6]. Statin could reduce expressions of adhesion molecules in monocyte [7], inhibit proliferation of macrophages in plaques [8], block CD4+ T-cell-mediated apoptosis [9], and suppress mRNA expressions of pro-inflammatory cytokines in PBMCs [10]. Given clinical studies have showed that statins would confer less risk reduction of cardiovascular diseases in individuals with high serum C-reactive protein (CRP) levels [11,12], anti-inflammatory treatment is emerging as a complement to lipid-lowering treatment in ASCVD patients [13]. Accordingly, how to reliably identify ASCVD patients with discernible inflammation in atheroma plaques could be fundamental to implementing anti-inflammatory treatment in clinical setting.

Serum CRP levels might, however, be a biomarker suboptimal to determine inflammation status of atheroma plaques, given that CRP levels could be promoted by immune and non-immune cells. In the Canakinumab Anti-Inflammatory Thrombosis Outcomes Study (CANTOS) recruiting patients with myocardial infarction and high CRP levels of ≥2 mg/dl at baseline, it was revealed that only 55% of patients receiving 150-mg canakinumab achieved CRP levels of <2 mg/dl at 3 months, thereby resulting in significant cardiovascular risk reduction, rather than those with achieved CRP levels of ≥2 mg/dl [14]. A meta-analysis has demonstrated that CVD risk reduction by statin treatment was not associated with achieved level of, or the reduction magnitude of CRP [15]. These findings might be partly because interleukin-6, a potent inducer for CRP production in hepatocytes, is secreted by immune cells [16,17], but also by non-immune cells, such as activated endothelial cells and smooth muscle cells [18,19].

microRNAs (miRNAs) are short non-coding RNAs for post-transcriptional regulations of gene expressions by binding mostly to the 3′ untranslated region of target mRNAs, thus leading to premature degradation and translation suppression of target mRNAs [20]. miRNAs have been reported to mediate statin-induced inflammation modulations [21–23], and, hence, might be feasible biomarkers for assessing inflammation status in immune cells. However, there is a paucity of evidence on comprehensive assessments for statin-induced miRNA alterations in immune cells. We here reported that miRNA alterations in hypercholesterolemic patients before and after receiving statin treatment were discovered using a microarray profiling method in PBMCs, which were an ensemble of immune cells involved in inflammation of atheroma plaques [6].

## Materials and methods

### Study design and participants

We conducted a sub-study of a multi-center, double-blinded, randomized clinical trial [24], whose study protocol was approved by the research ethics committee of the National Taiwan University Hospital (REC No. 201106075RC). A total of 34 patients, who were recruited in the National Taiwan University Hospital, participated this sub-study, after written informed consents were obtained. Of those, 19 patients received 2 mg pitavastatin daily, and the remaining did 10 mg atorvastatin daily for 12 weeks. Medical history and anthropometric measurements were procured on enrollment.

### Blood sampling and isolation of PBMCs

Blood samples were collected after overnight fasting at enrollment and 12 weeks. Laboratory measurements were carried out in a central laboratory, including total cholesterol, triglyceride, high-density-lipoprotein cholesterol (HDL-C), LDL-C, and high-sensitivity CRP. PBMCs were isolated using the Ficoll-Paque PLUS (GE Healthcare) density gradient centrifugation, and were frozen at −20°C for RNA extraction.

### RNA extraction and profiling miRNA transcriptome of PBMCs

After PBMCs were properly thawed, total RNA of PBMCs were purified using TRIzol® reagent (Invitrogen). Quantification of RNA was performed using a ND-1000 spectrophotometer (NanoDrop Technologies, Wilmington, DE). We then profiled miRNA alterations using the Affymetrix GeneChip® miRNA 3.0 Array (Affymetrix, Santa Clara, CA), which offered a total of 1733 human mature miRNA probe sets according to the miRBase database version 17. After being polyadenylated, RNAs were labeled using the FlashTag™ Biotin HSR RNA Labeling Kit. The biotin-labeled RNAs was hybridized onto microarrays at 48°C and 60 rpm for 16 h. Then, miRNA-hybridized microarrays were stained with phycoerythrin-conjugated streptavidin in a GeneChip® Fluidics Station 450. Finally, the fluorescence-stained microarrays were scanned for probe signal intensities in a GeneChip® Scanner 3000 7G. The average intensities of the spike-ins and background probe sets were plotted and compared for quality control; and all microarrays achieved the requirements of quality control. The microarray data have been submitted to the Gene Expression Omnibus (accession number: GSE152016).

### Selection and validation of miRNAs with stain-induced differential expressions

We applied three methods to identify miRNA candidates with statin-induced differential expression. After microarray intensity signals were pre-processed using the robust multi-array (RMA) method [25], the fold-change method and the Significance Analysis of Microarrays (SAM) method were used to select differentially expressed miRNA candidates [26]. The third method was that miRNA signals were fit into linear models for microarray (limma), after pre-processed using the variance stabilizing normalization (VSN) [27,28].

The selected miRNAs candidates were further validated using the quantitative polymerase chain reaction (qPCR) method. Total RNAs were analyzed according to the standard protocol of the miScript PCR System (QIAGEN), and SNORD68 was used as the endogenous control for normalization. miRNAs with differential expressions were defined if the microarray-measured fold changes of >1.50 or <0.67 were validated by qPCR.

### Biological inference of miRNA-modulated signaling pathways

The biological inference of miRNAs was initiated by identifying targeted genes, whose expressions might be modulated by miRNAs. The plausible links between targeted genes and miRNAs could be determined by well-established algorithms or experimental evidences. Based on dedicated database, signaling pathways were fitted by the targeted genes, and were scored and ranked using over-representation enrichment analysis. It was, therefore, inferred that biological pathways with higher ranking were more likely to be modulated by the miRNAs. The web-based miRSystem [29] and miTALOS [30] were platforms designed to incorporate the process of identifying targeted genes and pathway enrichment analysis. Accordingly, we applied the miRNAs with qPCR-validated differential expressions to miRSystem and miTALOS platforms to perform biological inference of miRNA-modulated signaling pathways. We used default settings of the miRSystem, while conducted the leukocyte-confined pathway analysis in the miTALOS platform. Besides, the normalized expressions of targeted genes in PBMCs were adopted from the Blood Atlas of the Human Protein Atlas database [31].

### Statistical analysis

Continuous variables were presented as mean and standard deviation; and categorical ones, as number and percentage, unless otherwise specified. Categorical variables were tested using the chi square test or Fisher exact test, where appropriate.

The microarray intensity data were pre-processed using the robust multi-array (RMA) or variance stabilizing normalization (VSN) method [25,32]. The RMA method consists of background correction, quantile normalization, logarithm transformation, and then median polish summarization [25]. Because of the dependence of the variance on the mean intensity, especially in the miRNAs with low signal intensities, the VSN method was proposed to estimate mean-independent variance based on an additive-multiplicative error model fitting generalized logarithm-transformed intensity data after affine calibration [32]. Fold changes of miRNA intensities were used to represent the alterations of miRNA expressions between before- and after-treatment. Given PMBCs were mostly in naïve status, fold changes of miRNAs could be lower in PMBCs than immune cells in atheroma plaques [33]. Accordingly, we defined miRNAs as being differentially expressed if fold change of >1.50 or <0.67, along with the *q* value or the false discovery rate of <0.05 for the correction of multiple comparisons [34]. Although miRNAs with differential expressions could be selected using the Student’s *t* test, the stability of *t* statisitc is subject to the small sample size and low expression levels. SAM and limma methods were recommended to address the inappropriateness of the Student’s *t* test by adjusting variance estimations in a nonparametric or parametric way, respectively. The SAM method calculated a statistic score, whose *q* value was derived according to permutation estimation [26]. The limma method used the empirical Bayes approach to estimate robust gene-specific variances of linear models for calculating a moderated *t* statistic, which followed a *t* distribution under null hypothesis [28]. Benjamini–Hochberg adjustment for multiple comparisons was used to control family-wise error rate in the limma method [35].

Correlations of post-treatment lipid and CRP changes with the SAM-derived fold changes of differentially expressed miRNAs were assessed after sex, age, and statin type were adjusted. To delineate the relationship of differentially expressed miRNAs with baseline CRP levels, we compared log-transformed fold changes among the CRP tertiles after adjusting for age, sex, and statin type. The statistical powers of the sample size in the present study were estimated to be greater than 0.95 using the SAM method, provided that truly changed genes with fold changes of >1.5 in paired samples were 500 or less [26]. The two-sided *P* value of less than 0.05 was considered statistically significant. The statistical analysis was performed using the SAS software, version 9.4. (SAS Institute, Cary, NC), and R software, version 3.6.1. (R Foundation for Statistical Computing, Vienna, Austria), along with the Bioconductor Packages [36].

## Results

Among the 34 hypercholesterolemic patients aged 63.7 ± 6.2 years (Table 1), there were 19 (55.9%) patients with stable coronary heart disease, 21 (61.8%) ones with Type 2 diabetes mellitus under oral hypoglycemic treatment, 32 (94.1%) ones with hypertension. And only 5 (14.7%) patients were current smokers (Supplementary Table S1). The proportions of patients with coronary heart disease, diabetes mellitus, hypertension, and smoking habit were not different between the pitavastatin and atorvastatin subgroups (*P* = 0.092, 0.16, 0.19, and 0.63, respectively).

**Table 1:** Clinical and laboratory characteristics before and after 12-week atorvastatin or pitavastatin treatment in patients with hypercholesterolemia

	Total		
	(*n*=34)		
	Before Tx	After Tx	*P*
Age, mean (SD), years	63.7 (6.2)		
Female, *n* (%)	7 (20.6)		
Body mass index, mean (SD), kg/m^2^	26.8 (3.7)		
Systolic blood pressure, mean (SD), mmHg	125.1 (8.5)		
Diastolic blood pressure, mean (SD), mmHg	76.4 (7.3)		
Medical history			
Coronary heart disease, *n* (%)	19 (55.9)		
Diabetes mellitus, *n* (%)	21 (61.8)		
Hypertension, *n* (%)	32 (94.1)		
Current smoker, *n* (%)	5 (14.7)		
Laboratory data			
Hemoglobin, mean (SD), g/dl	14.9 (1.1)	14.9 (1.1)	0.87
Platelet, mean (SD), 10^3^/μl	222.1 (59.0)	222.4 (59.3)	0.94
White blood cell, mean (SD), 10^3^/ml	5.9 (1.3)	6.3 (1.6)	0.042
Neutrophil, mean (SD), %	54.6 (8.2)	56.8 (8.2)	0.043
Lymphocyte, mean (SD), %	35.9 (7.4)	33.7 (7.0)	0.020
Monocyte, mean (SD), %	5.6 (1.2)	5.7 (1.5)	0.50
AST, mean (SD), U/l	29.3 (8.9)	29.2 (12.0)	0.95
ALT, mean (SD), U/l	34.4 (18.1)	34.8 (18.9)	0.90
CPK, mean (SD), U/l	132.3 (75.6)	206.4 (414.7)	0.29
Fasting glucose, mean (SD), mg/dl	117.1 (24.9)	122.6 (37.3)	0.12
HbA1c, mean (SD), %	6.7 (0.9)	6.6 (1.0)	0.61
CRP, mean (SD), mg/dl	0.15 (0.24)	0.19 (0.42)	0.66
Lipid profile			
Total cholesterol, mean (SD), mg/dl	236.4 (32.0)	169.4 (36.1)	<0.001
Triglyceride, mean (SD), mg/dl	169.8 (56.7)	127.4 (46.8)	<0.001
HDL-C, mean (SD), mg/dl	50.3 (11.3)	49.2 (11.5)	0.34
LDL-C, mean (SD), mg/dl	164.7 (28.4)	108.2 (37.1)	<0.001

Abbreviations: ALT, alanine aminotransferase; AST, aspartate aminotransferase; CPK, creatine phosphokinase; CRP, C-reactive protein; HDL-C, high-density-lipoprotein cholesterol; LDL-C, low-density-lipoprotein cholesterol; SD, standard deviation; Tx, treatment.

While the reduction percentage of the LDL-C level was 34.5 ± 17.3% after 12-week statin treatment, pitavastatin and atorvastatin treatment achieved similar post-treatment decrease in LDL-C levels (reduction percentage, 32.8 ± 17.5% versus 36.6 ± 17.5%; *P*=0.53). Among 34 patients, only one had CRP level of greater than 1 mg/dl (Table 1). Statin treatment did not significantly lower serum CRP level (pre-treatment, 0.15 ± 0.24 versus post-treatment, 0.19 ± 0.42 mg/dl; *P*=0.66) (Supplementary Table S1). Similarly, there was no difference in CRP changes between the pitavastatin or atorvastatin subgroup (*P* = 0.67, 0.34, respectively).

**Figure 1:**
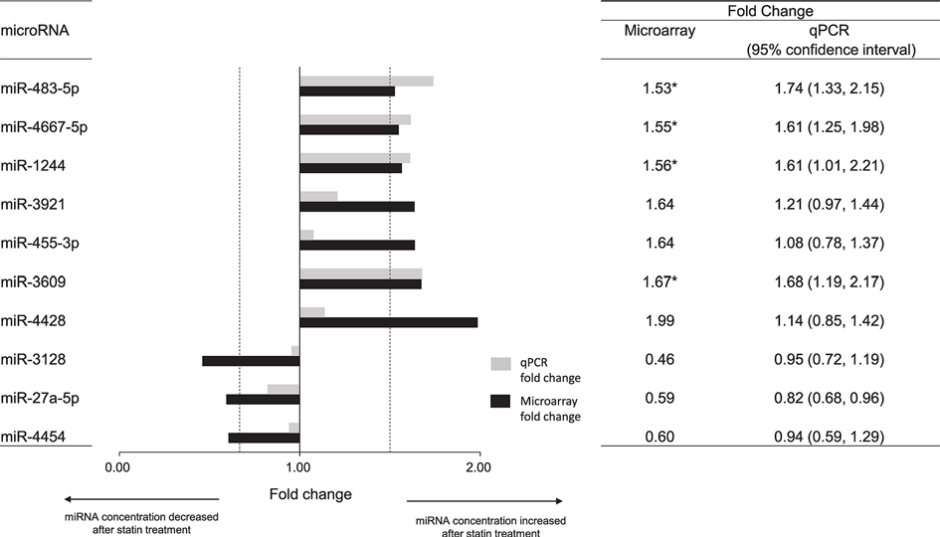
Statin-induced differential expressions of miRNAs in peripheral blood mononuclear cells (PBMC) miRNAome alterations were profiled using microarrays in 34 hypercholesterolemic patients before and after 12-week statin treatment. Fold changes were defined as the ratios of post-treatment to pre-treatment miRNA signal intensities. miRNAs were considered as having statin-induced differential expressions, if their microarray-profiled fold changes were >1.50 or <0.67 with quantitative polymerase chain reaction (qPCR) validation. * indicated the miRNAs with qPCR-validated fold changes of >1.50 or <0.67. Dotted vertical lines represented as fold changes of 1.50 and 0.67, respectively.

Based on microarray signal intensities, a total of 21 miRNAs candidates with fold changes of >1.50 or <0.67 were selected for qPCR validation. Among them, there were 10 miRNAs having the microarray fold changes in concordance with ones of qPCR validation, in which reference sequences were listed in the Supplementary Table S2. As shown in the Figure 1, we identified that statins induced differential expressions of miR-483-5p, miR-4667-5p, miR-1244, and miR-3609, with qPCR-validated fold changes of 1.74 (95% confidence interval, 1.33–2.15), 1.61 (1.25–1.98), 1.61 (1.01–2.21), and 1.68 (1.19–2.17), respectively.

**Figure 2:**
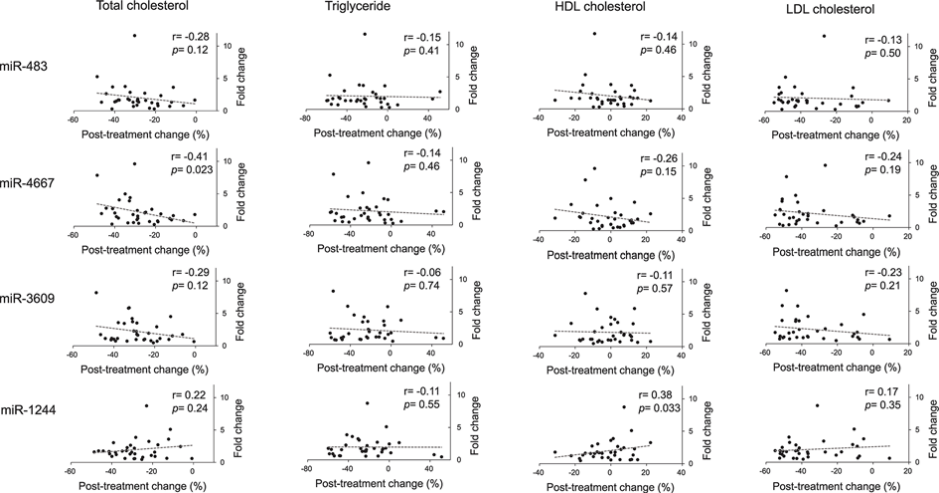
Associations between magnitudes of lipid changes and fold changes of the four miRNAs after statin treatment Correlations of serum lipid changes with fold changes of the four differentially expressed PBMC miRNAs in hypercholesterolemic patients receiving 12-week statin treatment. Lipid changes were defined as (post-treatment minus pre-treatment lipid level)/pre-treatment lipid level. Fold changes of miRNAs were the ratios of post-treatment to pre-treatment microarray-profiled signal intensities. Correlations were adjusted for sex, age, and statin type. Dotted lines represented linear regression lines.

**Figure 3:**
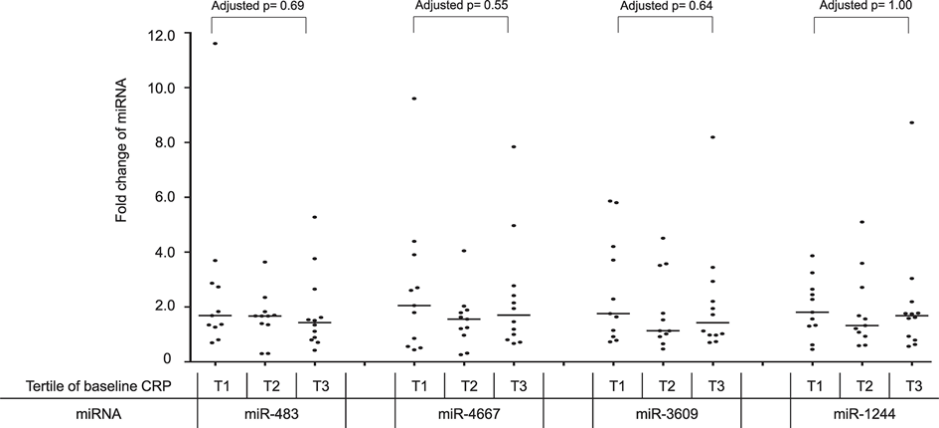
Alterations in statin-induced fold changes of the four PMBC-derived miRNAs across the tertiles of baseline serum CRP levels Comparing the statin-induced fold changes of the four differentially expressed PBMC miRNAs after 12-week statin treatment according to the tertiles of baseline CRP levels. The fold changes of miRNAs were defined as the ratios of post-treatment to pre-treatment miRNA signal intensities derived from microarray profiling. The comparisons were adjusted for sex, age, and statin type.

In the Figures 2 and 3, we explored that, after statin treatment, the fold changes of the four miRNAs would be related to magnitudes of lipid changes and baseline CRP levels, respectively. The Figure 2 showed the age-, sex-, and statin type-adjusted associations between lipids and fold changes of the four differentially expressed miRNAs. While the fold changes of miR-4667 and miR-1244 appeared to correlate with post-treatment changes of total cholesterol and HDL-C (adjusted *r* = −0.41 and 0.38, *P*=0.023 and 0.033, respectively), the associations were not found between the fold changes of miRNAs and LDL-C or triglyceride changes, suggesting that there were greater individual variations in statin-induced miRNA alterations than those in lipid changes. On the other hand, the fold changes of miRNAs were not different among the baseline CRP tertiles (Figure 3), and nor were correlated with post-treatment CRP changes (sex-, age, and statin type-adjusted correlation coefficient, *r* = −0.07, 0.001, −0.02, and −0.08; *P*=0.70, 0.97, 0.91, and 0.69, for miR-483-5p, miR-4667-5p, miR-3609, and miR-1244, respectively), probably because serum CRP levels could be affected by the IL-6 production of inflammatory and non-inflammatory cells.

**S**ignaling pathways were determined using miRSystem (Table 2A) and miTALOS (Table 2B) platforms, respectively, in the descending order of likelihood that some or all of the four differentially expressed miRNAs could be involved in modulation of biological signaling.

**Table 2:** Identification of potential signaling pathways modulated by the four microRNAs with statin-induced differential expressions according to the miRSystem (A) and the miTALOS (B) platforms dedicated to integrating the process of targeted gene exploration and pathway enrichment analysis

Database	Signaling pathway	Brief description of signaling pathway	Targeted genes	miRNAs	Ranking score
**(A) The most relevant ten pathways identified using the miRSystem platform**					
KEGG	Leishmaniasis	Disease-specific pathway	ELK1, MARCKSL1	miR-483	0.75
Pathway interaction database	PDGFR-β signaling pathway	PDGFR-β is a member of PDGF family, and could activate Ras to promote cell proliferation.	RHOA, ELK1	miR-483	0.626
Reactome	NGF signaling via TRKA from the plasma membrane	TRK signaling regulates cell proliferation and neuronal differentiation.	RHOA, ELK1	miR-483	0.609
KEGG	Focal adhesion	The biological processes regulate cell motility, proliferation, differentiation, survival, and gene expressions.	RHOA, ELK1	miR-483	0.527
Reactome	Signaling by neurotrophin receptor	Signaling networks relating to neuronal survival, differentiation, and plasticity.	RHOA, ELK1	miR-483	0.505
Pathway interaction database	Endogenous TLR signaling	A family of pattern recognition receptors are involved in innate and adaptive immunity, and could be triggered by endogenous ligands in inflammation conditions.	RHOA	miR-483	0.451
Reactome	G_βγ_ signaling through PI3Kγ	PI3Kγ is highly expressed in neutrophils, and is activated by membrane-bound G_βγ_.	RHOA	miR-483	0.451
Biocarta	BIOCARTA_RACCYCD_PATHWAY	Regulation of G1 to S transition involving Ras, Rac, Rho, and cyclin D1 expression.	RHOA	miR-483	0.446
Biocarta	BIOCARTA_EDG1_PATHWAY	Phospholipids as signaling intermediaries involving regulations of actin assembly, chemotaxis, proliferation and cell survival.	RHOA	miR-483	0.442
Biocarta	BIOCARTA_ERK_PATHWAY	Erk1/Erk2 MAPK signaling pathway to regulate growth, differentiation, and development.	ELK1	miR-483	0.438
Database	Signaling pathway	Brief description of signaling pathway	Targeted genes	corrected p value	
**(B) Pathways identified using the miTALOS platform**					
KEGG	TGF-β signaling pathway	Biological processes regulating cell growth, apoptosis, homeostasis, and differentiation.	TGFBR2, SMAD2, SMAD4, SAMD7, SMURF1, SMURF2, BMPR2, ACVR1B, ACVR2A, SP1, EP300, TNF, PPP2CA, MAPK1, RHOA	0.008	
WikiPathways	Physiological and pathological hypertrophy of the heart	Disease-specific signaling network	RHOA, MAPK1, FOS, IL6ST, PRKCE, PPP3CA, SPAG1	0.016	
WikiPathways	Mesodermal commitment pathway	Biological processes involving development	BMPR2, SMAD2, SMAD4, ACVR2A, PHF6, NLK, NFE2L2, TOX, C9orf72, WDCP, AEBP2, PARP8, ATP8B2, CCDC6, EMSY, EXT2, UBR5, SETD2, PPP2CA, PBX3	0.042	

Abbreviations: G_βγ_, β and γ subunits of G-protein; KEGG, Kyoto Encyclopedia of Genes and Genomes; MAPK, mitogen-activated protein kinase; NGF, nerve growth factor; PI3Kγ, phosphatidylinositol 3-kinase-γ; PDGF, platelet-derived growth factor; PDGFR, platelet-derived growth factor receptor; TGF-β, transforming growth factor β; TLR, Toll-like receptor

Among the four differentially expressed miRNAs, only miR-483 was identified by at least three of the miRSystem-integrated prediction programs. The Table 2 outlined the signaling pathways enriched by targeted genes of the differentially expressed miRNAs according to the analysis using the miRSystem and miTALOS platforms. In Table 2A and Supplementary Table S3, it was revealed that RHOA was the mostly expressed in PBMCs among the targeted genes of miR-483, and was the mostly identified among the enriched signaling pathways. As for the most relevant ten signaling identified by the miRSystem, the relevant pathways in the context of PBMCs included focal adhesion, endogenous Toll-like receptor (TLR) signaling, and intracellular signaling networks related to G_βγ_ protein, cyclin D1, and sphingosine 1-phosphate (Table 2B). While confined to the gene expressions of leukocytes, the miTALOS analysis indicated that transforming growth factor-beta (TGF-β) signaling pathway was the most relevant to statin-induced miRNA alterations.

## Discussions

In hypercholesterolemic patients receiving 12-week statin treatment, we found that statin treatment could induce miRNA alterations in PBMCs consisting of immune cells involved in the inflammation reactions of atheroma plaques. Four miRNAs of miR-483-5p, miR-4667-5p, miR-3609, and miR-1244 with statin-induced increased expressions were determined using unbiased microarray profiling along with qPCR validations. The four miRNAs were not associated with post-treatment serum LDL-C reduction, nor with the baseline level or post-treatment reduction in serum CRP. Exploring the biological relevance of the four miRNAs with statin-induced differential expressions in PBMCs, we revealed that signaling pathways involving inflammation reactions were relevant to the post-treatment miRNA alterations, including RhoA-related focal adhesion and TGF-β signaling. Our findings indicated that the four miRNAs might be involved in statin-induced inflammation modulations, and thus be feasible biomarkers for assessing inflammation status in response to statin treatment.

### Statin-induced miRNA regulations of RhoA GTPase expression

The mechanism for statin-induced anti-inflammatory effects has been proposed that isoprenoid intermediates of cholesterol biosynthesis are collectively suppressed by statins, thus reducing the post-translational prenylation of Rho and Rac GTPases [12]. Prenylation with the isoprenoid intermediates, including farnesylpyrophosphate (FPP) and geranylgeranylpyrophosphate (GGPP) [37], facilitates Rho and Rac GTPases attached onto cell membranes, where these GTPases are normally functional for signaling transductions. RhoA binds the downstream effector, Rho-associated coiled-coil-containing protein kinase (ROCK), and then alters cytoskeleton through myosin light chain or cofilin phosphorylation [38,39], whereby focal adhesion and transendothelial migration of monocytes and T-cells are regulated [40,41].

The relationships between miRNAs and RhoA signaling in circulating immune cells are yet to be elucidated, though it has been evidenced that miRNAs were involved in post-transcriptional regulations of RhoA signaling in non-immune cells. Prior studies have reported that miR-31, miR-125a-3p, miR-133a, miR-155, and miR-185 decreased RhoA expressions in osteoclasts, lung cancer cells, cardiomyocytes, breast cancer cells, and colorectal cancer cells, respectively [42–46]. In the present study, we discovered the association between statin treatment and miR-483-5p expressions. Given that RhoA is essential to signaling of focal adhesion and transendothelium migration of immune cells, statin-induced increase in miR-483-5p expression might repress RhoA-mediated inflammatory reactions.

### Statin-induced miRNA regulations of context-dependent TGF-β signaling

TGF-β exerts context-dependent effects on innate and adaptive immune responses via canonical and non-canonical pathways, thereby leading to dual pro-inflammatory and anti-inflammatory roles in the progression and resolution of atheroma plaques. The canonical pathway is mediated in the Smad-dependent way [47], while the non-canonical signaling is mediated in Smad-independent ways, such as inter-connections with RhoA/ROCK signaling pathway [48]. TGF-β is a pro-inflammatory chemoattractant to induce migration of monocytes and dendritic cells [49,50]. On the other hand, the anti-inflammatory effects of TGF-β has been implicated because the disruption of TGF-β signaling in T-cells could lead to atherosclerosis progression [51]. However, when incorporating different interleukin cytokines, TGF-β could facilitate or inhibit CD4+ T-cells differentiation into distinct T-cell subsets, thereby promoting or resolving inflammation in atheroma plaques. In the presence of TGF-β, CD4+ T-cells differentiation are inhibited into Th1 and Th2 cells; but are promoted into Th9, Th17, and Treg when coupled with IL-4, IL-6, or IL-2, respectively [52]. In general, Th1, Th9, and Th17 cells mediate pro-inflammatory reactions, whileTh2 and Treg cells do anti-inflammatory ones, though some evidence showed that Th17 cells might have dual effects on plaque stability [53]. Besides, the effects of TGF-β are inhibitory and pro-apoptotic for CD8+ T-cells, NK cells, and B cells [54,55].

Statins have been reported to modulate TGF-β expressions and signaling in immune and non-immune cells. In cultured THP-1 cells, TGF-β expression and production were up-regulated by pravastatin in a dose-dependent manner [56]. A clinical study found that, in hypercholesterolemic patients treated with daily 40 mg pravastatin for 4–6 weeks, post-treatment serum TGF-β levels were elevated, but also that isolated peripheral blood monocytes increased TGF-β expression and production, independently of post-treatment lipid changes [57]. However, there is a paucity of evidence regarding how statins modulate TGF-β signaling. Our findings revealed an *in-silico* link between TGF-β signaling and statin-induced miRNA alterations in PBMCs. miR-483-5p, miR-4667-5p, miR-3609, and miR-1244 might regulate the expressions of extensive elements in the TGF-β signaling pathway, including the expressions of TGF-β receptors, Smad proteins, Smad inhibitory proteins, transcriptional factors, and non-Smad proteins, such as RhoA. More studies are required to determine the responses of PBMCs when TGF-β signaling is modulated by those miRNAs in diverse pathophysiological settings.

### Study limitations

Some limitations in our study should be reminded. First, PBMCs are limited to delineate statin-induced miRNAome alterations in distinct immune cell subsets. Interindividual variations on proportions of immune cell types in PBMCs might make it less feasible to detect miRNAs with low expression amount and high variability [33]. Second, miRNA extraction from PBMCs appeared to be more technique-demanding than from plasma, resulting in the concern on the feasibility in clinical practice. However, changes of circulating miRNAs could not be comparable to those of intracellular miRNAs, partly because not all miRNAs in immune cells are secreted in exosomes [58]. Third, as for high-throughput detection of miRNAs, microarray profiling is known to be less specific than RNA sequencing [59]. Given that the purpose of our study was to explore the associations between statin treatment and miRNA-regulated inflammatory responses in immune cells, microarray profiling could be adequate to detect statin-induced miRNA alterations [59]. Fourth, confining pathway analysis to healthy leukocytes in the miTALOS platform might increase the biological relevance at the expanse of exploring unexpected connections in hypercholesterolemia-primed PBMCs. Considering that, we combined the results of biological relevance derived from an unconfined and a confined pathway analysis using the miRSystem and the miTALOS platforms, respectively.

## Perspectives

1. Given that miRNAs mediate in statin-induced anti-inflammation, and that CRP might be suboptimal to assess inflammation status in ASCVD patients, evidence remains lacking on comprehensive profiling of statin-induced miRNAome alterations in circulating immune cells.
2. We profiled miRNAome alterations in hypercholesterolemic patients before and after statin treatment, and discovered that statin treatment led to the increased expressions of miR-483-5p, miR-4667-5p, miR-1244, and miR-3609 in PBMCs. And the four miRNAs with statin-induced differential expressions were involved in RhoA and transforming growth factor-β signaling pathways, probably modulating transendothelium migration and differentiations of immune cells.
3. Our findings implicated that miRNAs of PBMCs might be feasible to assess inflammation status, but also be therapeutic potentials to modulate inflammation in ASCVD patients.

## Supplementary Material

Supplementary Tables S1-S3

## Abbreviations

ASCVD: atherosclerotic cardiovascular disease
CRP: C-reactive protein
LDL-C: low-density lipoprotein cholesterol
PBMC: peripheral blood mononuclear cell
qPCR: quantitative polymerase chain reaction

## References

[1] Weber C. and Noels H. (2011) Atherosclerosis: current pathogenesis and therapeutic options. Nat. Med. 17, 1410–1422 10.1038/nm.2538 22064431

[2] (2018) Global, regional, and national age-sex-specific mortality for 282 causes of death in 195 countries and territories, 1980-2017: a systematic analysis for the Global Burden of Disease Study 2017. Lancet 392, 1736–1788 10.1016/S0140-6736(18)32203-7 30496103 PMC6227606

[3] Group, W.C.R.C.W. (2019) World Health Organization cardiovascular disease risk charts: revised models to estimate risk in 21 global regions. Lancet Glob. Health 7, e1332–e1345 10.1016/S2214-109X(19)30318-3 31488387 PMC7025029

[4] Silverman M.G., Ference B.A., Im K., Wiviott S.D., Giugliano R.P., Grundy S.M. et al. (2016) Association Between Lowering LDL-C and Cardiovascular Risk Reduction Among Different Therapeutic Interventions: A Systematic Review and Meta-analysis. JAMA 316, 1289–1297 10.1001/jama.2016.13985 27673306

[5] Tousoulis D., Psarros C., Demosthenous M., Patel R., Antoniades C. and Stefanadis C. (2014) Innate and adaptive inflammation as a therapeutic target in vascular disease: the emerging role of statins. J. Am. Coll. Cardiol. 63, 2491–2502 10.1016/j.jacc.2014.01.054 24613322

[6] Kleiveland C.R. (2015) Peripheral Blood Mononuclear Cells. In The Impact of Food Bioactives on Health: in vitro and ex vivo models(Verhoeckx K., Cotter P., Lopez-Exposito I., Kleiveland C., Lea T., Mackie A., Requena T., Swiatecka D. and Wichers H., eds), pp. 161–167, Springer Copyright 2015, Cham (CH) 29787039

[7] Wójciak-Stothard B., Williams L. and Ridley A.J. (1999) Monocyte Adhesion and Spreading on Human Endothelial Cells Is Dependent on Rho-regulated Receptor Clustering. J. Cell Biol. 145, 1293–1307 10.1083/jcb.145.6.1293 10366600 PMC2133155

[8] Tang J., Lobatto M.E., Hassing L., van der Staay S., van Rijs S.M., Calcagno C. et al. (2015) Inhibiting macrophage proliferation suppresses atherosclerotic plaque inflammation. Sci. Adv. 1, e1400223 10.1126/sciadv.1400223 26295063 PMC4539616

[9] Sato K., Nuki T., Gomita K., Weyand C.M. and Hagiwara N. (2010) Statins reduce endothelial cell apoptosis via inhibition of TRAIL expression on activated CD4 T cells in acute coronary syndrome. Atherosclerosis 213, 33–39 10.1016/j.atherosclerosis.2010.03.034 20430391 PMC2914144

[10] Cerda A., Rodrigues A.C., Alves C., Genvigir F.D., Fajardo C.M., Dorea E.L. et al. (2015) Modulation of adhesion molecules by cholesterol-lowering therapy in mononuclear cells from hypercholesterolemic patients. Cardiovasc. Ther. 33, 168–176 10.1111/1755-5922.12126 25903419

[11] Ridker P.M., Danielson E., Fonseca F.A., Genest J., Gotto A.M. Jr, Kastelein J.J. et al. (2008) Rosuvastatin to prevent vascular events in men and women with elevated C-reactive protein. N. Engl. J. Med. 359, 2195–2207 10.1056/NEJMoa0807646 18997196

[12] Oesterle A., Laufs U. and Liao J.K. (2017) Pleiotropic Effects of Statins on the Cardiovascular System. Circ. Res. 120, 229–243 10.1161/CIRCRESAHA.116.308537 28057795 PMC5467317

[13] Ridker P.M., Everett B.M., Thuren T., MacFadyen J.G., Chang W.H., Ballantyne C. et al. (2017) Antiinflammatory Therapy with Canakinumab for Atherosclerotic Disease. N. Engl. J. Med. 377, 1119–1131 10.1056/NEJMoa1707914 28845751

[14] Ridker P.M., MacFadyen J.G., Everett B.M., Libby P., Thuren T. and Glynn R.J. (2018) Relationship of C-reactive protein reduction to cardiovascular event reduction following treatment with canakinumab: a secondary analysis from the CANTOS randomised controlled trial. Lancet 391, 319–328 10.1016/S0140-6736(17)32814-3 29146124

[15] Zhang X.L., Lan R.F., Zhang X.W., Xu W., Wang L., Kang L.N. et al. (2019) Association Between Baseline, Achieved, and Reduction of CRP and Cardiovascular Outcomes After LDL Cholesterol Lowering with Statins or Ezetimibe: A Systematic Review and Meta-Analysis. J. Am. Heart Assoc. 8, e012428 10.1161/JAHA.119.012428 31411090 PMC6759897

[16] Gabay C. (2006) Interleukin-6 and chronic inflammation. Arthritis Res. Ther. 8, S3 10.1186/ar1917 16899107 PMC3226076

[17] Rezaie-Majd A., Maca T., Bucek R.A., Valent P., Muller M.R., Husslein P. et al. (2002) Simvastatin reduces expression of cytokines interleukin-6, interleukin-8, and monocyte chemoattractant protein-1 in circulating monocytes from hypercholesterolemic patients. Arterioscler. Thromb. Vasc. Biol. 22, 1194–1199 10.1161/01.ATV.0000022694.16328.CC 12117737

[18] Greenwood J. and Mason J.C. (2007) Statins and the vascular endothelial inflammatory response. Trends Immunol. 28, 88–98 10.1016/j.it.2006.12.003 17197237 PMC3839264

[19] Ito T., Ikeda U., Shimpo M., Ohki R., Takahashi M., Yamamoto K. et al. (2002) HMG-CoA reductase inhibitors reduce interleukin-6 synthesis in human vascular smooth muscle cells. Cardiovasc. Drugs Ther. 16, 121–126 10.1023/A:1015701415588 12090904

[20] Treiber T., Treiber N. and Meister G. (2019) Regulation of microRNA biogenesis and its crosstalk with other cellular pathways. Nat. Rev. Mol. Cell Biol. 20, 5–20 10.1038/s41580-018-0059-1 30728477

[21] Xie W., Li P., Wang Z., Chen J., Lin Z., Liang X. et al. (2014) Rosuvastatin May Reduce the Incidence of Cardiovascular Events in Patients with Acute Coronary Syndromes Receiving Percutaneous Coronary Intervention by Suppressing miR-155/SHIP-1 Signaling Pathway. Cardiovasc. Ther. 32, 276–282 10.1111/1755-5922.12098 25319951

[22] Satoh M., Tabuchi T., Minami Y., Takahashi Y., Itoh T. and Nakamura M. (2012) Expression of let-7i is associated with Toll-like receptor 4 signal in coronary artery disease: Effect of statins on let-7i and Toll-like receptor 4 signal. Immunobiology 217, 533–539 10.1016/j.imbio.2011.08.005 21899916

[23] Frostegard J., Zhang Y., Sun J., Yan K. and Liu A. (2016) Oxidized Low-Density Lipoprotein (OxLDL)-Treated Dendritic Cells Promote Activation of T Cells in Human Atherosclerotic Plaque and Blood, Which Is Repressed by Statins: microRNA let-7c Is Integral to the Effect. J. Am. Heart Assoc. 5, e003976 10.1161/JAHA.116.003976 27650878 PMC5079044

[24] Liu P.Y., Lin L.Y., Lin H.J., Hsia C.H., Hung Y.R., Yeh H.I. et al. (2013) Pitavastatin and Atorvastatin double-blind randomized comPArative study among hiGh-risk patients, including thOse with Type 2 diabetes mellitus, in Taiwan (PAPAGO-T Study). PLoS One 8, e76298 10.1371/journal.pone.0076298 24098467 PMC3788128

[25] Irizarry R.A., Hobbs B., Collin F., Beazer-Barclay Y.D., Antonellis K.J., Scherf U. et al. (2003) Exploration, normalization, and summaries of high density oligonucleotide array probe level data. Biostatistics 4, 249–264 10.1093/biostatistics/4.2.249 12925520

[26] Tusher V.G., Tibshirani R. and Chu G. (2001) Significance analysis of microarrays applied to the ionizing radiation response. Proc. Natl. Acad. Sci. U. S. A. 98, 5116–5121 10.1073/pnas.091062498 11309499 PMC33173

[27] Smyth G.K. (2004) Linear models and empirical bayes methods for assessing differential expression in microarray experiments. Stat. Appl. Genet. Mol. Biol. 3, 1–26 Article 3 10.2202/1544-6115.1027 16646809

[28] Ritchie M.E., Phipson B., Wu D., Hu Y., Law C.W., Shi W. et al. (2015) limma powers differential expression analyses for RNA-sequencing and microarray studies. Nucleic Acids Res. 43, e47 10.1093/nar/gkv007 25605792 PMC4402510

[29] Lu T.P., Lee C.Y., Tsai M.H., Chiu Y.C., Hsiao C.K., Lai L.C. et al. (2012) miRSystem: an integrated system for characterizing enriched functions and pathways of microRNA targets. PLoS ONE 7, e42390 10.1371/journal.pone.0042390 22870325 PMC3411648

[30] Preusse M., Theis F.J. and Mueller N.S. (2016) miTALOS v2: Analyzing Tissue Specific microRNA Function. PLoS ONE 11, e0151771 10.1371/journal.pone.0151771 26998997 PMC4801359

[31] Uhlen M., Karlsson M.J., Zhong W., Tebani A., Pou C., Mikes J. et al. (2019) A genome-wide transcriptomic analysis of protein-coding genes in human blood cells. Science 366, eaax9198 10.1126/science.aax9198 31857451

[32] Huber W., von Heydebreck A., Sultmann H., Poustka A. and Vingron M. (2002) Variance stabilization applied to microarray data calibration and to the quantification of differential expression. Bioinformatics 18, S96–S104 10.1093/bioinformatics/18.suppl_1.S96 12169536

[33] Mohr S. and Liew C.C. (2007) The peripheral-blood transcriptome: new insights into disease and risk assessment. Trends Mol. Med. 13, 422–432 10.1016/j.molmed.2007.08.003 17919976

[34] Storey J.D. and Tibshirani R. (2003) Statistical significance for genomewide studies. Proc. Natl. Acad. Sci. U. S. A. 100, 9440–9445 10.1073/pnas.1530509100 12883005 PMC170937

[35] Benjamini Y. and Hochberg Y. (1995) Controlling the False Discovery Rate: A Practical and Powerful Approach to Multiple Testing. J. Royal Statistical Soc. Ser. B (Methodological). 57, 289–300 10.1111/j.2517-6161.1995.tb02031.x

[36] Huber W., Carey V.J., Gentleman R., Anders S., Carlson M., Carvalho B.S. et al. (2015) Orchestrating high-throughput genomic analysis with Bioconductor. Nat. Methods 12, 115–121 10.1038/nmeth.3252 25633503 PMC4509590

[37] Goldstein J.L. and Brown M.S. (1990) Regulation of the mevalonate pathway. Nature 343, 425–430 10.1038/343425a0 1967820

[38] Noma K., Oyama N. and Liao J.K. (2006) Physiological role of ROCKs in the cardiovascular system. Am. J. Physiol. Cell Physiol. 290, C661–C668 10.1152/ajpcell.00459.2005 16469861 PMC2692274

[39] Sit S.T. and Manser E. (2011) Rho GTPases and their role in organizing the actin cytoskeleton. J. Cell Sci. 124, 679–683 10.1242/jcs.064964 21321325

[40] Worthylake R.A., Lemoine S., Watson J.M. and Burridge K. (2001) RhoA is required for monocyte tail retraction during transendothelial migration. J. Cell Biol. 154, 147–160 10.1083/jcb.200103048 11448997 PMC2196864

[41] Heasman S.J., Carlin L.M., Cox S., Ng T. and Ridley A.J. (2010) Coordinated RhoA signaling at the leading edge and uropod is required for T cell transendothelial migration. J. Cell Biol. 190, 553–563 10.1083/jcb.201002067 20733052 PMC2928012

[42] Care A., Catalucci D., Felicetti F., Bonci D., Addario A., Gallo P. et al. (2007) MicroRNA-133 controls cardiac hypertrophy. Nat. Med. 13, 613–618 10.1038/nm1582 17468766

[43] Huang B., Luo W., Sun L., Zhang Q., Jiang L., Chang J. et al. (2013) MiRNA-125a-3p is a negative regulator of the RhoA-actomyosin pathway in A549 cells. Int. J. Oncol. 42, 1734–1742 10.3892/ijo.2013.1861 23525486

[44] Kong W., Yang H., He L., Zhao J.J., Coppola D., Dalton W.S. et al. (2008) MicroRNA-155 is regulated by the transforming growth factor beta/Smad pathway and contributes to epithelial cell plasticity by targeting RhoA. Mol. Cell. Biol. 28, 6773–6784 10.1128/MCB.00941-08 18794355 PMC2573297

[45] Mizoguchi F., Murakami Y., Saito T., Miyasaka N. and Kohsaka H. (2013) miR-31 controls osteoclast formation and bone resorption by targeting RhoA. Arthritis Res. Ther. 15, R102 10.1186/ar4282 24004633 PMC3978447

[46] Liu M., Lang N., Chen X., Tang Q., Liu S., Huang J. et al. (2011) miR-185 targets RhoA and Cdc42 expression and inhibits the proliferation potential of human colorectal cells. Cancer Lett. 301, 151–160 10.1016/j.canlet.2010.11.009 21186079

[47] Batlle E. and Massague J. (2019) Transforming Growth Factor-beta Signaling in Immunity and Cancer. Immunity 50, 924–940 10.1016/j.immuni.2019.03.024 30995507 PMC7507121

[48] Luo K. (2017) Signaling Cross Talk between TGF-beta/Smad and Other Signaling Pathways. Cold Spring Harb. Perspect. Biol. 9, a022137 10.1101/cshperspect.a022137 27836834 PMC5204325

[49] Wahl S.M., Hunt D.A., Wakefield L.M., McCartney-Francis N., Wahl L.M., Roberts A.B. et al. (1987) Transforming growth factor type beta induces monocyte chemotaxis and growth factor production. Proc. Natl. Acad. Sci. U. S. A. 84, 5788–5792 10.1073/pnas.84.16.5788 2886992 PMC298948

[50] Sato K., Kawasaki H., Nagayama H., Enomoto M., Morimoto C., Tadokoro K. et al. (2000) TGF-β1 Reciprocally Controls Chemotaxis of Human Peripheral Blood Monocyte-Derived Dendritic Cells Via Chemokine Receptors. J. Immunol. 164, 2285 10.4049/jimmunol.164.5.2285 10679062

[51] Robertson A.K., Rudling M., Zhou X., Gorelik L., Flavell R.A. and Hansson G.K. (2003) Disruption of TGF-beta signaling in T cells accelerates atherosclerosis. J. Clin. Invest. 112, 1342–1350 10.1172/JCI18607 14568988 PMC228445

[52] Sanjabi S., Oh S.A. and Li M.O. (2017) Regulation of the Immune Response by TGF-beta: From Conception to Autoimmunity and Infection. Cold Spring Harb. Perspect. Biol. 9, a022236 10.1101/cshperspect.a022236 28108486 PMC5453394

[53] Gistera A., Robertson A.K., Andersson J., Ketelhuth D.F., Ovchinnikova O., Nilsson S.K. et al. (2013) Transforming growth factor-beta signaling in T cells promotes stabilization of atherosclerotic plaques through an interleukin-17-dependent pathway. Sci. Transl. Med. 5, 196ra100 10.1126/scitranslmed.3006133 23903754

[54] Tamayo E., Alvarez P. and Merino R. (2018) TGFbeta Superfamily Members as Regulators of B Cell Development and Function-Implications for Autoimmunity. Int. J. Mol. Sci. 19, 3928–3945 10.3390/ijms19123928 PMC6321615 30544541

[55] Viel S., Marcais A., Guimaraes F.S., Loftus R., Rabilloud J., Grau M. et al. (2016) TGF-beta inhibits the activation and functions of NK cells by repressing the mTOR pathway. Sci. Signal 9, ra19 10.1126/scisignal.aad1884 26884601

[56] Baccante G., Mincione G., Di Marcantonio M.C., Piccirelli A., Cuccurullo F. and Porreca E. (2004) Pravastatin up-regulates transforming growth factor-beta1 in THP-1 human macrophages: effect on scavenger receptor class A expression. Biochem. Biophys. Res. Commun. 314, 704–710 10.1016/j.bbrc.2003.12.150 14741692

[57] Porreca E., Di Febbo C., Baccante G., Di Nisio M. and Cuccurullo F. (2002) Increased transforming growth factor-beta(1) circulating levels and production in human monocytes after 3-hydroxy-3-methyl-glutaryl-coenzyme a reductase inhibition with pravastatin. J. Am. Coll. Cardiol. 39, 1752–1757 10.1016/S0735-1097(02)01857-0 12039487

[58] Montecalvo A., Larregina A.T., Shufesky W.J., Stolz D.B., Sullivan M.L., Karlsson J.M. et al. (2012) Mechanism of transfer of functional microRNAs between mouse dendritic cells via exosomes. Blood 119, 756–766 10.1182/blood-2011-02-338004 22031862 PMC3265200

[59] Pritchard C.C., Cheng H.H. and Tewari M. (2012) MicroRNA profiling: approaches and considerations. Nat. Rev. Genet. 13, 358–369 10.1038/nrg3198 22510765 PMC4517822

