# Supplementary material for: Statin-induced microRNAome alterations modulating inflammation pathways of peripheral blood mononuclear cells in patients with hypercholesterolemia
Source: Biosci Rep. 2020 Sep 21;40(9):BSR20201885. doi: 10.1042/BSR20201885 (PMC7507599; doi:10.1042/BSR20201885)
Supplement: Supplementary Tables S1-S3 [file BSR-2020-1885_supp.pdf]

Supplemental Table 1. Baseline clinical features and laboratory measurements before and after statin treatment stratified by statin types.

	Pitavastatin (n=19)		Atorvastatin (n=15)	
	Before Tx	After Tx	Before Tx	After Tx
Age, mean (SD), years	65.7 (5.5)		61.1 (6.2)	
Female, n (%)	6 (31.6)		1 (6.7)	
Body mass index, mean (SD), kg/m^2	26.1 (3.2)		27.6 (4.3)	
Systolic blood pressure, mean (SD), mmHg	126.7 (8.4)		123.1 (8.5)	
Diastolic blood pressure, mean (SD), mmHg	76.7 (7.8)		76.0 (6.8)	
Medical history				
Coronary heart disease, n (%)	8 (42.1)		11 (73.3)	
Diabetes mellitus, n (%)	14 (73.7)		7 (46.7)	
Hypertension, n (%)	19 (100.0)		13 (86.7)	
Current smoker, n (%)	2 (10.5)		3 (20.0)	
Laboratory data				

Hemoglobin, mean (SD), g/dL	14.7 (1.1)	14.7 (1.2)	15.1 (1.2)	15.1 (1.1)
Platelet, mean (SD), 10^3/□L	215.7 (57.8)	218.8 (60.8)	230.2 (61.5)	226.9 (59.1)
White blood cell, mean (SD), 10^3/mL	5.6 (1.2)	5.8 (1.5)	6.3 (1.3)	6.9 (1.6)
Neutrophil, mean (SD), %	54.7 (8.9)	55.5 (8.8)	54.5 (7.4)	58.5 (7.4)
Lymphocyte, mean (SD), %	35.8 (7.7)	34.8 (7.2)	36.1 (7.2)	32.2 (6.6)
Monocyte, mean (SD), %	5.8 (1.5)	6.1 (1.7)	5.4 (0.9)	5.3 (1.0)
AST, mean (SD), U/L	29.1 (10.0)	28.7 (10.2)	29.7 (7.5)	29.8 (14.3)
ALT, mean (SD), U/L	31.0 (17.0)	31.7 (17.4))	38.7 (19.1)	38.7 (20.6)
CPK, mean (SD), U/L	127.8 (75.8)	249.6 (552.2)	138.0 (77.7)	151.7 (86.9)
Fasting glucose, mean (SD), mg/dL	123.3 (27.2)	130.4 (43.8)	109.4 (19.7)	112.8 (25.1)
HbA1c, mean (SD), %	6.8 (0.8)	6.7 (0.9)	6.5 (1.1)	6.5 (1.2)
CRP, mean (SD), mg/dL	0.10 (0.17)	0.06 (0.09)	0.22 (0.30)	0.34 (0.60)
Lipid profile				
Total cholesterol, mean (SD), mg/dL	233.9 (31.8)	172.8 (32.0)	239.6 (33.2)	165.1 (41.5)
Triglyceride, mean (SD), mg/dL	152.3 (40.1)	118.8 (42.4)	191.9 (67.6)	138.3 (51.3)
HDL cholesterol, mean (SD), mg/dL	50.9 (10.5)	52.0 (12.1)	49.4 (12.6)	45.7 (10.0)

LDL cholesterol, mean (SD), mg/dL	164.7 (29.4)	110.3 (35.2)	164.6 (28.0)	105.7 (40.4)

Abbreviations: ALT: alanine aminotransferase; AST: aspartate aminotransferase; CPK: creatine phosphokinase; CRP: C-reactive protein; HDL-C: high-density-lipoprotein cholesterol; LDL-C: low-density-lipoprotein cholesterol; SD: standard deviation; Tx: treatment.

Supplemental Table 2. Reference sequences of microRNAs used for quantitative polymerase chain reaction (qPCR) validation.

Microarray-selected miRNA	Reference sequence
hsa-miR-483-5p	AAGACGGGAGGAAAGAAGGGAG
hsa-miR-4667-5p	ACUGGGGAGCAGAAGGAGAACC
hsa-miR-1244	AAGUAGUUGGUUUGUAUGAGAUGGUU
hsa-miR-3921	UCUCUGAGUACCAUAUGCCUUGU
hsa-miR-455-3p	GCAGUCCAUGGGCAUAUACAC
hsa-miR-3609	CAAAGUGAUGAGUAAUACUGGCUG
hsa-miR-4428	CAAGGAGACGGGAACAUGGAGC
hsa-miR-3128	UCUGGCAAGUAAAAAACUCUCAU
hsa-miR-27a-5p	AGGGCUUAGCUGCUUGUGAGCA
hsa-miR-4454	GGAUCCGAGUCACGGCACCA

Abbreviations: miRNA, microRNA; qPCR, quantitative polymerase chain reaction.

Supplemental Table 3. Targeted genes of miR-483 and their normalized expressions in peripheral blood mononuclear cells (PBMCs) according to the miRSystem platform and the Human Protein Atlas

Targeted gene	Gene description	Normalized expression in PBMC
RHOA	ras homolog family member A	151.5
CTDSPL2	CTD (carboxy-terminal domain, RNA polymerase II, polypeptide A) small phosphatase like 2	11.2
ELK1	ELK1, member of ETS oncogene family	6.3
NDRG2	NDRG family member 2	4.0
PRPF4B	pre-mRNA processing factor 4B	3.9
CXXC5	CXXC finger protein 5	3.4
CPNE5	copine V	2.2
NUDT8	nudix (nucleoside diphosphate linked moiety X)-type motif 8	2.1
FAM160B2	family with sequence similarity 160, member B2	2.1
SLC12A5	solute carrier family 12 (potassium/chloride transporter), member 5	1.3
MARCKSL1	MARCKS-like 1	1.2

NFIX	nuclear factor I/X (CCAAT-binding transcription factor)	0.5
SOX11	SRY (sex determining region Y)-box 11	0.5
HAND2	heart and neural crest derivatives expressed 2	0.0
PAX2	paired box 2	0.0
LDLRAD2	low density lipoprotein receptor class A domain containing 2	0.0
RNF165	ring finger protein 165	0.0
